# Faster But Less Precise: Expectation Enhances Response Speed While Reducing Sensory Fidelity

**DOI:** 10.1523/JNEUROSCI.0154-26.2026

**Published:** 2026-06-22

**Authors:** Ziyue Hu, Dominic M. D. Tran, Reuben Rideaux

**Affiliations:** School of Psychology, The University of Sydney, Camperdown 2050, Australia

**Keywords:** EEG, prediction, selective attention, vision

## Abstract

The brain's remarkable ability to process continuous sensory inputs with adaptive efficiency—balancing flexibility while minimizing metabolic cost—is thought to rely on predictive mechanisms that generate and update internal models that leverage statistical regularities in the environment. However, it remains unclear whether this efficiency arises from prioritizing reliable, expected events or informative, unexpected ones, as they offer complementary adaptive advantages. To isolate genuine expectation effects, we combined electroencephalography (EEG), pupillometry, and behavioral measures in a paradigm that independently manipulated task relevance (selective attention) and stimulus predictability, while minimizing stimulus repetition at identical spatial locations to control for low-level adaptation. Human participants (both sexes) responded faster and more accurately to expected events, which was enhanced when attention was engaged; however, these events were reproduced with lower precision, independent of attention. Feature-specific neural decoding revealed prestimulus effects of attention and poststimulus effects of expectation, with no interaction between the two. Attention increased decoding accuracy, while expectation reduced accuracy. The reduced representational fidelity for expected events appeared rapidly (∼100–200 ms after stimulus onset) and correlated with individual differences in perceptual precision. Collectively, our findings indicate two complementary processes that define how the brain leverages redundancy in the environment: an early (prestimulus) mechanism, which supports rapid motor responses to expected events and is mediated by attention, and a later (poststimulus) process, which dampens sensory responses to expected events and is unaffected by attention.

## Significance Statement

The brain must process vast amounts of sensory information efficiently while remaining flexible enough to detect important changes. Competing theories propose that the brain saves energy either by prioritizing expected events or by enhancing responses to surprising ones, but experimental evidence has been inconsistent. Our findings show that the brain uses both strategies, each serving a different purpose. Expected events are processed quickly to support rapid, accurate actions, whereas expected events are also encoded with reduced precision, prioritizing unexpected events to update internal models and guide future behavior. By cleanly separating expectation from attention and low-level adaptation, we provide a unified explanation of how the brain balances efficiency with adaptability across multiple timescales.

## Introduction

The brain is continuously confronted with a torrent of sensory information, yet it achieves the remarkable feat of processing this stream with high fidelity and minimal metabolic cost. One influential proposal is that this efficiency arises from predictive mechanisms that leverage statistical regularities in the environment to generate internal models of upcoming sensory input (Rao and Ballard, 1999). By anticipating what is likely to occur, the brain can reduce redundant processing, allocate resources to behaviorally important signals, and update internal models when predictions fail. However, despite the centrality of prediction-based accounts, a persistent debate remains: does neural efficiency arise because the brain prioritizes expected events or because it prioritizes unexpected ones?

Expected events—those that are frequent, stable, and statistically reliable—are thought to support efficient processing by enabling the brain to preactivate stimulus-specific templates and reduce the computational burden of encoding predictable information. Numerous studies have attributed attenuated neural responses to expected stimuli as signatures of efficient coding and fulfilled predictions (Kok et al., et al., 2012a; Teufel et al., 2018; Yon et al., 2018). From this perspective, prediction serves to economize neural activity by sharpening expected representations and minimizing the metabolic cost associated with redundant input.

Conversely, unexpected events—those that deviate from prior expectations—are thought to elicit enhanced neural responses because they provide the greatest information gain. Prediction errors, which signal discrepancies between expected and observed input, enable internal models to be refined for future encounters with the environment (Tang et al., 2018, 2023; Lee and Rideaux, 2025; Rideaux et al., 2025b). A growing body of behavioral and neurophysiological work has reported heightened sensitivity, improved perceptual fidelity, and enhanced neural encoding for unexpected events. From this perspective, efficient processing arises because the brain devotes resources to informative signals that drive adaptive updating.

In principle, these accounts need not be mutually exclusive. Theoretical work has proposed that efficient perception may rely on multiple processes operating at different timescales and hierarchical levels (Press et al., 2020; Teufel and Fletcher, 2020). However, empirical evidence has been difficult to reconcile due to substantial methodological challenges. Chief among these is the confounding influence of low-level adaptation: many paradigms rely on stimulus repetition or feature cuing to induce expectations, unintentionally conflating prediction-based modulations with sensory habituation (Feuerriegel, 2024). Studies that rigorously eliminate repetition effects often report null expectation effects, leading some researchers to argue that many findings attributed to predictive processing may instead reflect simpler cell-level phenomena.

A second challenge concerns the relationship between expectation and selective attention. Both processes bias sensory processing toward certain inputs, and many experimental designs inadvertently entangle them (Alink and Blank, 2021). Whether attention gates, amplifies, reverses, or operates independently of expectation remains under active debate (Kok et al., 2012b; Jiang et al., 2013; Auksztulewicz and Friston, 2016; Smout et al., 2019; Lee and Rideaux, 2025). As a result, it is unclear whether expectation effects observed in behavior or neural activity reflect predictive mechanisms per se or the modulatory influence of attentional allocation.

To address these limitations, we developed an experimental paradigm designed to (1) robustly establish expectations without repeating stimuli at identical spatial locations, (2) independently manipulate selective attention and stimulus predictability, and (3) permit simultaneous measurement of behavioral performance, perceptual precision, autonomic signatures of surprise, and neural representational fidelity.

Our results show that expectation supports adaptive efficiency through two dissociable mechanisms. Expected events yielded faster and more accurate responses—an effect amplified by attention—suggesting a mechanism that supports behavioral optimization for interaction with the environment (i.e., rapidly responding to an upcoming expected event). In contrast, expected events were reproduced with lower perceptual precision and elicited less precise neural representations shortly after stimulus onset, independent of attentional demands. These neural–behavioral correspondences demonstrate that representational dampening of expected events reduce perceptual fidelity, minimizing metabolic expenditure and optimizing prediction of future events through model updating that prioritizes unexpected events.

## Materials and Methods

### Participants

We recruited 40 $\left(M_{\mathrm{age}}=20.9,\;N_{\mathrm{male}}=12\right)$ naive participants with normal or corrected-to-normal vision (assessed with a standard Snellen eye chart) from the University of Sydney and the community sample. They were compensated either two-unit credits or 40AUD for their participation of a 2 h experiment. This sample size was consistent with recent work employing the same neural decoding technique (Harrison et al., 2023; Rideaux, 2024; Hu et al., 2025; Buhmann et al., 2026). Ethics covering the current study was approved by the University of Sydney's Psychology Low Risk Human Ethics Committee.

### Apparatus

The study was set up in a testing room isolated as much as possible from external light, sound, and electromagnetic interference. Task stimuli were presented on mid-gray background overlaid with a low-contrast, 1/*f^2^* luminance texture to create a naturalistic and comfortable viewing experience for the participants. The experiment was performed on a display monitor (ASUS VG248QE 144 Hz 24″ 1,920 × 1,080 HDMI Gaming Monitor) via MATLAB R2020a (The MathWorks) and Psychtoolbox (v3.0, http://psychtoolbox.org/; Pelli, 1997). As instructed, participants placed their head on a forehead-chin rest, and we were thus able to maintain the viewing distance at 70 cm across participants with the screen subtended to 41.47° × 23.3°.

### Stimuli, task, and procedure

For each trial, single black Gaussian blob (*σ* = 0.3°, contrast = 0.2) was presented 4° from fixation randomly along an invisible circular path (0–360°) for a consistent duration (200 ms). A centered white fixation dot (radius = 0.25°) remained visible throughout to minimize eye movements. Participants were tasked with making a speeded binary judgment of whether each target (blob) appeared to the left versus right (via pressing key “z” and “/”) or above versus below fixation (via pressing key “b” and “t”). This assignment defined one cardinal plane (horizontal or vertical) as task relevant and the other as task irrelevant, counterbalanced across participants. Upon responding, the next target was presented following a 250 ms delay. Expectancy was manipulated independently along each reference plane by biasing the probability that successive targets repeated (R) or alternated (A) relative to the previous target location (75% vs 25%). This means that, on each trial, the next target's quadrant was probabilistically determined by the last-trial target's location and the assigned bias condition, after which its precise position was randomly sampled within that quadrant. Critically, these biases altered the probability of spatial repetition or alternation, rather than the absolute spatial location (e.g., left or right). For example, for participants assigned to the RA condition, each target stimulus would stay in the same vertical location with 75% likelihood while switching to the other horizontal location with 75% likelihood in reference to the preceding target. This resulted in 2 (task-relevant plane: horizontal or vertical) × 2 (vertical bias: A or R) × 2 (horizontal bias: A or R) between-subjects conditions, counterbalanced across participants. On a random set (10%) of trials, following the binary judgment, the target was replaced by a green blob (randomly positioned on the circular path). Participants were instructed to rotate the green blob to match the previous target's location as precisely as possible using the same keys as in the binary task (“z”/“/” or “b”/“t” for clockwise/counterclockwise) and confirm their response by pressing the spacebar. There were no time limits for the reproduction task, and these trials were subject to the same probabilities as the standard trials. Each participant completed 30 blocks with 100 trials each, with feedback on their response time and accuracy at the end of each block. Before the experiment, they received both verbal and on-screen instructions to maintain their attention on the central fixation point, respond to black blobs as quickly and accurately as possible, and reproduce green blobs as precisely as possible.

### EEG acquisition and preprocessing

This study employed 64-channel active Ag/AgCl electrodes arranged according to the international 10–20 standard (Oostenveld and Praamstra, 2001), with an online reference set at FCz. EEG signals were recorded at a 1,024 Hz sampling rate using a 24 bit A/D conversion and a BrainVision actiCAP system (Brain Products). We performed EEG data preprocessing with EEGLAB version 2,021.1 (Delorme and Makeig, 2004). Data were downsampled to 256 Hz, high-pass filtered at 0.1 Hz to remove slow drifts, and low-pass filtered at 45 Hz to remove high-frequency noise. We further performed independent component analysis (ICA) with the SASICA pipeline to identify and remove components associated with ocular and other artifacts, e.g., eyeblinks (Chaumon et al., 2015). The cleaned data were epoched from −200 to 500 ms relative to the target stimulus' onset. A small fraction of epochs failed to be recorded due to hardware faults (<1% of data from four participants). We only included the 24 rear (occipital and parietal) sensors in the analyses (1) to reduce the influence of signals produced by eye movements, blinks, and nonsensory cortices, (2) for consistency with similar previous decoding studies (Rideaux, 2024; Buhmann et al., 2026), and (3) to improve decoding accuracy by restricting sensors to those that carried the most spatial position information.

### Pupillometry

Pupil diameter changes were recorded with an EyeLink 1000 system (SR Research) sampling at 1 kHz and monitored live by the experimenter. Pupillometry recordings were epoched to a duration of −500 to 1,500 ms around the target stimulus presentation. Pupillometry data was preprocessed by removing blinks (±100 ms buffer window), removing outliers (median absolute deviation >2.5), interpolated, bandpass filtered with a first-order Butterworth filter (high pass, 0.0156 Hz; low pass, 1 Hz), *z*-scored, epoched, and downsampled to 125 Hz, respectively. These epochs were then grouped according to expectancy, and the difference between expected and unexpected targets $\left(\Delta_{\mathrm{pupil}\;\mathrm{size}}=\mathrm{expected}-\mathrm{unexpected}\;\right)$ was calculated for each individual.

### Mixture modeling of reproduction task

In the reproduction task, we recorded participants' reproduction error, defined as the angular difference between the actual and reported target locations on the circular path. To disentangle genuine replication-based responses from random guesses, we fit a mixture model to each participant's data, consistent with previous work (Bays et al., 2009; Hu et al., 2025). Guess responses were modeled as a uniform distribution of errors, indicating no relation to the target location, whereas accurate reproductions were captured by a von Mises distribution centered on the true target. The concentration parameter of the model (κ) was interpreted as indicative of participants' recall precision. Since the distribution of individual precision was positively skewed, we applied a logarithmic transformation normalize it (lg κ).

### Neural decoding of stimulus location

To investigate how neural representations of spatial location varied as a function of expectancy, we applied an inverted encoding model to decode the target's location from EEG data, similar to previous work (Brouwer and Heeger, 2011; Tang et al., 2018; Harrison et al., 2023; Rideaux, 2024). Data were separately analyzed according to expectancy and task relevance, including both training and testing. There were a higher number of targets in the expected condition, so we matched the number of trials by randomly sampling data from this condition (without replacement) and averaging the results across 50 iterations. We first nominated a forward model that can hypothetically characterize the EEG activity responding to the target stimulus' location (an angular estimate). The parameters of this model were then inverted to reconstruct stimulus location from observed neural activity. Model performance was assessed using 10-fold cross-validation: in each iteration, 90% of trials trained the model and the remaining 10% tested decoding accuracy. Note, training and testing was performed separately at each time point. Consistent with prior studies (Tang et al., 2018; Smout et al., 2019), higher decoding accuracy was interpreted as more precise neural representations. Specifically, the forward model comprised five hypothetical spatial-tuning channels with preferred angular spatial location linearly spaced between 0 and 360°, each defined by a half-wave-rectified sinusoid raised to the fourth power; EEG activity on each trial was modeled as a weighted sum of these channels:

B=WC+E,(1)

where **B** stands for the EEG recordings (*m* sensors × *n* representations), **W** is an estimated weight matrix (*m* sensors × 5 channels), **C** (5 channels × *n* representations) is the hypothesized channel responses, and **E** represents residual errors.

To obtain weights that best reconstructed channel activity from EEG signals with minimized error, we adapted modified inverted encoding model procedures from recent M/EEG work (Mostert et al., 2015; Kok et al., 2017) to include noise covariance regularization, improving estimation accuracy given correlated signals across neighboring electrodes. Both **B** and **C** were mean-centered across trials for each sensor and channel. The model was trained on a subset of data within a 10-fold cross-validation framework, resulting in a response vector $c_{\mathrm{train},i}$ for each channel ***i***, based on the training trials $\left(n_{\mathrm{train}}\right)$. Channel-specific weights $\left(w_{i}\right)$ were then calculated using least squares estimation:

$$w_{i}=(c_{\mathrm{train},i}c_{\mathrm{train},i}^{T}{)}^{-1}c_{\mathrm{train},i}^{T}B_{\mathrm{train}},\;(2)$$

such that, $B_{\mathrm{train}}$ (*m* sensors × *n* representations) stands for the training dataset of the EEG recordings. Following this, the optimal spatial filter $v_{i}$ for reconstructing channel *i* activity was then estimated as follows:

$$v_{i}=\frac{{\tilde{\Sigma }}_{i}^{-1}w_{i}}{w_{i}^{T}{\tilde{\Sigma }}_{i}^{-1}w_{i}},\;(3)$$

where ${\tilde{\Sigma }}_{i}$ denotes the regularized noise covariance matrix for channel *i*. To effectively control for noise caused by correlated neighboring sensors, we estimated and incorporated the noise covariance into the filter computation. This noise suppression was further improved through shrinkage regularization, using an analytically derived optimal shrinkage parameter (Mostert et al., 2015), yielding a regularized covariance matrix ${\tilde{\Sigma }}_{i}$ via the following:

$${\hat{\Sigma }}_{i}=\frac{1}{n_{\mathrm{train}}-1}\varepsilon_{i}\varepsilon_{i}^{T},\;(4)$$

$$\varepsilon_{i}=B_{\mathrm{train}}-w_{i}c_{\mathrm{train},i},\;(5)$$

where $n_{\mathrm{train}}$ stands for the size of training set (i.e., number of trials in the training dataset). Spatial filters $v_{i}$ from all five location channels were combined into one general channel filter matrix **V** (*m* sensors × 5 channels), which was then used to reconstruct channel responses in the test set:

$$C_{\mathrm{test}}=V^{T}B_{\mathrm{test}}.\;(6)$$

We then decoded stimulus location for each trial by converting the reconstructed channel responses into polar form as follows:

$$z=c\;\cdot e^{i\varphi },\;(7)$$

where c denotes the vector of channel responses and φ stands for the vector of preferred angles for the channels. Following this, we continued to compute the estimated stimulus location (angular estimate) using the following:

$$\hat{\theta }=\arg(z),\;(8)$$

Decoding accuracy was defined as the correspondence between decoded and presented stimulus angles (Kok et al., 2017) and estimated by projecting the decoding-stimulus differences onto a vector oriented at $0^{\circ }$:

r^θ=Re[R_],R_=1n∑j=1nexp(i(θ^j−θ)).(9)

Based on this, we further computed the time-resolved difference in decoding accuracy between expected and unexpected stimuli $\left(\Delta_{\mathrm{decoding}}\right)$ for each participant and correlated it with their recall precision difference $\left(\Delta_{\mathrm{precision}}\right)$.

To test whether the prestimulus effect of task relevance was due to expectation, we separated the data into task-relevant expected and unexpected trials and then tested the expected data on a model trained on the unexpected data. Cross-validation was not used, as we trained and tested on separate data.

### Data reanalysis

We reanalyzed behavioral data from a previously published study (Lee and Rideaux, 2025). Experiment 1 of this study used the same task as the current study, with the exception that no global biases were implemented; that is, targets appeared at all locations with equal probability and there was no correlation between the location of subsequent targets. Because target locations were randomly determined, expectations in this experiment could not be formed and therefore could not be satisfied or violated. This formed the basis of the baseline data for which the behavioral data collected in the current study were compared.

### Statistical analysis

Wilcoxon signed rank tests (i.e., nonparametric *z*-tests; Wilcoxon, 1945) were conducted to compare the difference between expected and unexpected trials (Δ=expected−unexpected) for the response time and accuracy in the binary task and recall precision in the reproduction task. Wilcoxon rank sum tests were conducted to compare the difference between data in the current experiment and data reanalyzed from a previously published experiment (Lee and Rideaux, 2025). Mixed ANOVA tests were used to assess effects of bias and task relevance on behavioral measures. For neural and pupillometry time-series data, Type I error due to multiple comparisons across time was controlled using cluster-based permutation testing (Maris and Oostenveld, 2007). Each analysis involved multiple time point comparisons per test with 5,000 permutation samples and a cluster-forming alpha of 0.05. For each permutation sample, condition labels were randomly shuffled, and the relevant statistical test for each measure was recomputed at every time point. Specifically, we performed Wilcoxon *z*-tests for pupillometry data $\left(\Delta_{\mathrm{pupil}\;\mathrm{size}}=\mathrm{expected}-\mathrm{unexpected}\right)$ (Wilcoxon, 1945), paired *t* tests for neural decoding accuracy comparisons between expected and unexpected targets, repeated-measures ANOVA for testing for effects of expectation and task relevance, and Pearson's correlation coefficients for neurobehavioral mapping. Time points that had *p* < 0.05 were grouped into temporally adjacent clusters. The sum of respectively, *z*-, *t*-, or *r*-to-*z*–transformed values within each cluster defined the cluster's mass. The largest cluster masses from the 5,000 permutation samples formed the null distribution, against which the observed cluster masses were compared. Clusters exceeding the 95th percentile of the null distribution were deemed significant after cluster-level correction. This approach controls the weak familywise error rate while maintaining sensitivity to effects that unfold over time in autocorrelated neural and pupillometry data (Maris and Oostenveld, 2007; Groppe et al., 2011). Two-tailed tests were used for all analyses except the neurobehavioral analysis, where a right-tailed test was used to detect the presence of a positive relationship between neural and behavioral indices.

### Data and code availability

The raw data and analysis code are publicly available at the following repository: https://doi.org/10.17605/OSF.IO/NEKP7

## Results

**Figure 1.**
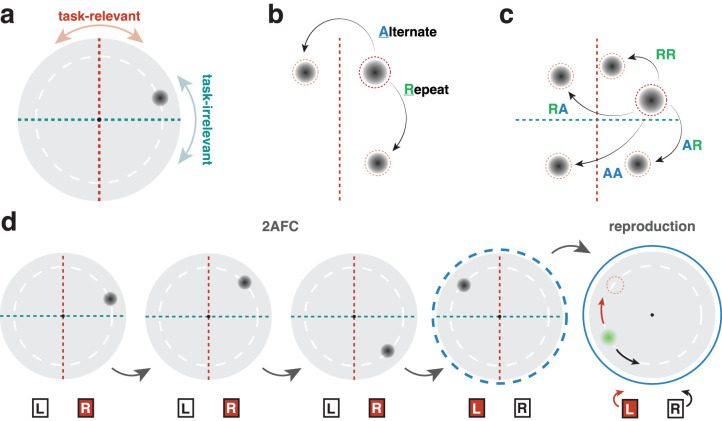
Experimental design. ***a***, Black Gaussian blobs were presented at random angular positions along an invisible circular path (white dashed circle). One cardinal plane was assigned as task relevant (red; binary task), leaving the other as task irrelevant (teal); task relevance was counterbalanced between the two cardinal planes across participants. ***b***, ***c***, Expectancy was manipulated by biasing the probability that blobs repeated (R; green) or alternated (A; blue) along each axis relative to the previous target (75% vs 25%), resulting in four spatial-transition possibilities (RR, RA, AR, AA). For example, in the RA condition, each target was more likely to repeat along the vertical plane but alternate along the horizontal plane, relative the preceding target. However, target locations were randomly selected within each quadrant to minimize low-level adaption induced by repetition. ***d***, In each trial, participants were tasked with making a speeded binary judgment of the target location relative to a cardinal plane (e.g., left/right of the fixation dot); on random subset (10%) of trials, they were subsequently instructed to reproduce the target location (blue dashed circle) by rotating a (green) probe blob clockwise or counterclockwise using key presses.

Recent debates have questioned how the brain's circuit-level design principles and higher-level expectancy effects respectively contribute to neural efficiency (den Ouden et al., 2023, 2025; Harrison et al., 2023; Feuerriegel, 2024; Hu et al., 2025; Rideaux et al., 2025a; Westerberg et al., 2025) and whether selective attention, i.e., the top-down prioritization of task-relevant inputs, conditionally modulates the instantiation or expression of these expectancy effects (Smout et al., 2019; Alink and Blank, 2021; Bouwkamp et al., 2025; Lee and Rideaux, 2025). Here we employed a paradigm that can simultaneously (1) establish robust expectancy, (2) control for low-level sensory adaptation, and (3) dissociate the effects of selective attention (Lee and Rideaux, 2025) to accurately characterize how expectation supports efficient neural processing. We asked participants to view single Gaussian blobs appearing at random angular positions along a circular path centered on the fixation dot (Fig. 1*a*). For each trial, they performed a speeded binary task judging whether each blob appeared to the left versus right or above versus below the fixation (Fig. 1*d*). This task assignment defined one cardinal axis (vertical or horizontal) as task relevant, leaving the other as task irrelevant (Fig. 1*a*,*d*, horizontal as task-relevant plane); the designation of relevance was counterbalanced between two planes across participants. Expectancy was manipulated by varying the probability that target locations repeated or alternated relative to the previous target (75% vs 25%, counterbalanced; Fig. 1*b*,*c*). For example, targets might often repeat along the vertical plane while alternating along the horizontal plane (Fig. 1*c*, RA). This design thus minimized low-level adaptation by avoiding repetitive stimulus presentation at identical spatial locations, while dissociating selective attention from expectancy through the independent manipulation of task relevance and stimulus probability. After the binary response, participants were tasked to precisely reproduce the spatial location of the target stimulus as an index of perceptual fidelity on a random 10% subset of trials (Fig. 1*d*). We recorded participants' pupil diameter changes via eye tracking and their neural responses via electroencephalography (EEG) and quantified neural fidelity of (un)expected targets via multivariate decoding (inverted encoding modeling; Brouwer and Heeger, 2009).

### Physiological validation and effect of expectancy on perceptual fidelity

**Figure 2.**
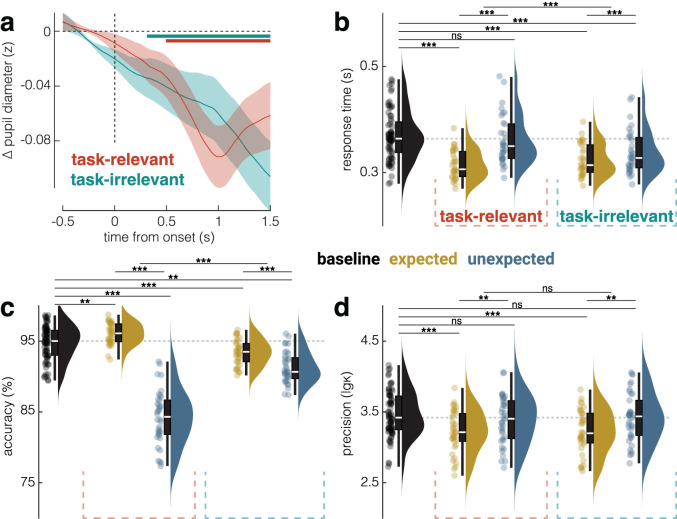
The effect of expectation on perceptual performance. ***a***, Normalized pupil size difference $(\Delta_{\mathrm{pupil}\;\;\mathrm{size}}=\mathrm{expected}-\mathrm{unexpected})\;$ as a function of time from target onset, along task-relevant and task-irrelevant planes. Shaded areas indicate ±1 SEM. Horizontal bars indicate cluster-corrected periods when $\Delta_{\mathrm{pupil}\;\mathrm{size}}$ significantly differs from zero. Violin plots show the distribution of participants' (***b***) response time and (***c***) accuracy in the binary task, and (***d***) precision for the reproduction task across task-relevant (red) and task-irrelevant (teal) planes, separately for expected (yellow) and unexpected (blue) targets. The black violin plot shows reanalyzed data (*n* = 79) from Experiment 1 of Lee and Rideaux (2025), where the same task was used, but targets appeared with equal probability, and serves as a baseline of no expectation; the gray dashed line is provided to support comparison with the baseline data. The median is indicated by boxplots within each violin and dots indicate individual participant data points. Significance (p<0.001,<0.01) is marked with *** and **, respectively, while “ns” denotes nonsignificant differences.

We quantified expectancy effects as the difference (Δ=expected−unexpected) in performance between relatively frequent (expected) and infrequent (unexpected) stimuli. Expectancy was defined by the biased probability of repeat and alternate trials, separately along task-relevant and -irrelevant planes (Fig. 1*b*,*c*). In line with previous work, we tested whether our expectancy manipulation induced pupil dilation, an autonomic physiological signature associated with expectancy violation (Hu et al., 2025; Lee and Rideaux, 2025; Rideaux et al., 2025b). Analysis of pupil diameter revealed significantly larger dilations for unexpected compared with expected stimulus locations $\left(\Delta_{\mathrm{pupil}\;\mathrm{size}}<0\right)$ along both task-relevant and task-irrelevant planes, peaking at ∼1 s poststimulus onset (Fig. 2*a*). This latency was consistent with previous studies (Preuschoff et al., 2011; Lee and Rideaux, 2025; Rideaux et al., 2025b).

Having found evidence supporting the effectiveness of our expectancy manipulation through physiological measures, we next examined its influence on participants' behavioral performance. Response time (RT) and accuracy (proportion of correct binary judgments) were measured in the binary task. For the reproduction task, we recorded the angular differences between the actual and reported target locations, with mixture modeling applied to estimate individuals' perceptual precision (i.e., the fidelity with which participants reproduced the target's spatial location). Expected events elicited faster and more accurate responses along both task-relevant (response time: *z*_(40)_ = 5.51, *p* = 3.57 × 10^−10^; accuracy: *z*_(40)_ = 5.51, *p* = 3.57 × 10^−10^) and task-irrelevant planes (response time: *z*_(40)_ = 4.06, *p* = 4.92 × 10^−7^; accuracy: *z*_(40)_ = 3.80, *p* = 1.42 × 10^−4^), with this advantage being stronger when the relative location was task-relevant (response time: *z*_(35)_ = 5.07, *p* = 4.00 × 10^−9^; accuracy: *z*_(39)_ = 5.44, *p* = 5.26 × 10^−10^; Fig. 2*b*,*c*). In contrast, expected events were reproduced with lower precision in both planes (task-relevant: *z*_(40)_ = 3.06, *p* = 0.002; task-irrelevant: *z*_(40)_ = 3.10, *p* = 0.001), and this disadvantage for expected events did not differ significantly as a function of task relevance (*z*_(40)_ = 0.18, *p* = 0.87; Fig. 2*d*).

The differences in performance between expected and unexpected targets could be due to effects of satisfying or violating expectation, or a combination of both. To determine the directionality of the effects, we reanalyzed a previously published dataset (*n* = 79) from an experiment that employed the same design, with the exception that targets were equally likely to be presented at all locations (Lee and Rideaux, 2025, their Experiment 1). This data provides a baseline measure, where target locations were uncorrelated across time. We found that both expected and unexpected events were responded to faster than those with no expectation, regardless of task relevance (expected-relevant: *z*_(102)_ = 5.82, *p* < 0.001; expected-irrelevant: *z*_(102)_ = 5.05, *p* < 0.001; unexpected-irrelevant: *z*_(103)_ = 3.43, *p* = 6.00 × 10^−4^), with the exception of unexpected events in the task relevant condition (unexpected-relevant: *z*_(102)_ = 1.21, *p* = 0.227), which did not differ significantly. Similarly, along the task irrelevant plane, both expected and unexpected events were responded to less accurately than those with no expectations (expected-irrelevant: *z*_(109)_ = 3.05, *p* = 0.002; unexpected-irrelevant: *z*_(108)_ = 5.88, *p* < 0.001). However, along the task relevant plane, we found a bidirectional effect such that expected events were responded to more accurately while unexpected events produced less accurate response (expected-relevant: *z*_(110)_ = 2.59, *p* = 0.001; unexpected-relevant: *z*_(108)_ = 8.25, *p* < 0.001). In contrast to response time and accuracy, which were influenced by both satisfaction and violation of expectation, we found that recall precision was selectively reduced for expected events, while unexpected events were recalled with equal fidelity to those without expectation (expected-relevant: *z*_(108)_ = 3.44, *p* = 6.0 × 10^−4^; unexpected-relevant: *z*_(108)_ = 1.01, *p* = 0.313; expected-irrelevant: *z*_(108)_ = 3.40, *p* = 6.0 × 10^−7^; unexpected-irrelevant: *z*_(108)_ = 0.54, *p* = 0.591). These findings indicate the presence of two distinct mechanisms: one that supports rapid and accurate motor responses to expected events and is mediated by attention and another that suppresses the encoding fidelity of expected events and is not mediated by attention.

**Figure 3.**
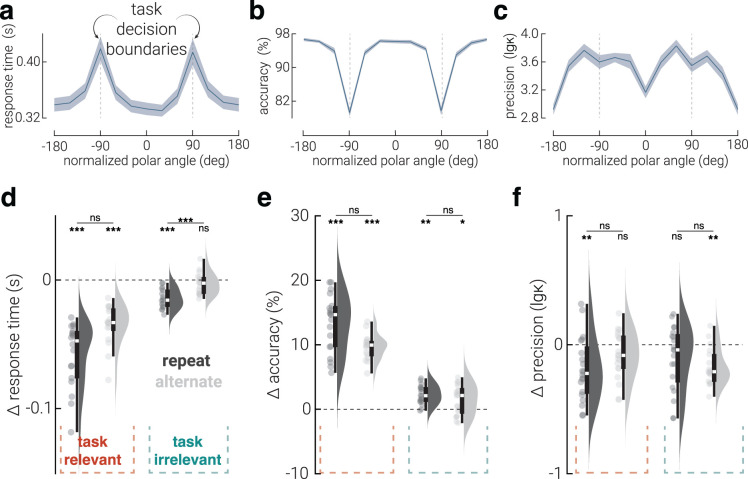
Effects of task demands and local spatiotemporal proximity on behavioral performance. ***a***, Response time and (***b***) accuracy in the binary task, and (***c***) precision for the reproduction task, as a function of distance from the binary task decision boundary. ***d***, ***f***, Violin plots showing the distribution of participant's performance difference (Δ=expected−unexpected) for (***d***) response time, (***e***) accuracy, and (***f***) precision across task-relevant (red) and -irrelevant (teal) planes, separately for participants with either repeating (dark gray) or alternating (light gray) target biases. The median is indicated by boxplots within each violin and dots indicate individual participant data points. Significance (p<0.001,<0.01) is marked with *** and **, respectively, while “ns” denotes nonsignificant differences.

We found that attention, as manipulated through task relevance, interacted with expectation when determining the speed and accuracy of participants' binary responses. In contrast, it appeared to have no impact of the on effect of expectation on recall precision. As a sanity check, we assessed the extent to which attention influenced behavior, independent of expectation, by calculating response time, accuracy, and precision, relative to the binary task decision boundary. If attention influences recall precision, we would expect to observe changes in responses around the boundary, where the task was most difficult and thus would motivate more attentional engagement. As expected, response times were slowest and accuracy poorest around the decision boundary (Fig. 3*a*,*b*). Critically, precision was highest around the decision boundary and considerably worse around the orthogonal boundary (Fig. 3*c*). These results show that demands of the binary task effectively influenced precision on the reproduction task and confirm that the absence of an interaction between attention and expectation on precision was not for lack of attentional engagement.

To establish sensory predictions, we manipulated global spatiotemporal regularities within the stimulus, i.e., probabilistic biases for repeating or alternating target locations. However, previous work has shown that local spatiotemporal properties can influence response times, accuracy, and recall precision (Lee and Rideaux, 2025). In the previous analyses, we controlled for local spatiotemporal influences by counterbalancing expectancy biases across both directions (repetition and alternation). To test for interactions between global and local properties, we calculated the difference in response time, accuracy, and precision between (globally) expected and unexpected targets, separately for participants in repeating and alternating bias conditions. Echoing the results for task relevance, we found a significant main effect of bias on response time and accuracy, but not precision (response time: *F*_(1,29)_ = 13.1, *p* = 0.001; accuracy: *F*_(1,31)_ = 15.5, *p* = 4.0 × 10^−4^; precision: *F*_(1,35)_ = 0.2, *p* = 0.655). That is, responses were faster and more accurate for repeating biases, consistent with previous work (Lee and Rideaux, 2025). Critically, by counterbalancing these biases between participants and collapsing across this dimension, we mitigated the influence of this effect in the main analyses.

### Effect of expectancy on neural fidelity

**Figure 4.**
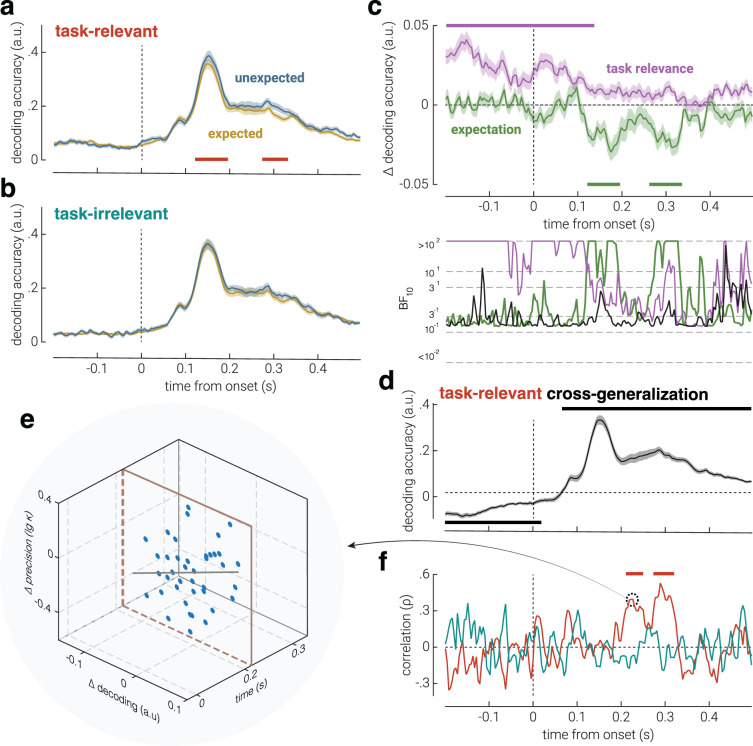
Effects of expectancy and attention on neural fidelity and neurobehavioral mapping. Decoding accuracy for the target location of expected (yellow) and unexpected (blue) targets along (***a***) task-relevant and (***b***) task-irrelevant planes, as a function of time. Note, decoding accuracy is above chance prior to stimulus onset because the location of targets is correlated, due to the expectancy manipulation. That is, the location of target *n* predicts that of target *n* + 1, so the decoder can (weakly) predict the location of the upcoming target from the residual activity of the previous target. Horizontal bars in ***a*** indicate cluster-corrected periods when decoding accuracy differs significantly. ***c***, The difference in decoding accuracy between expectation $(\Delta_{\mathrm{decoding}}=\mathrm{expected}-\mathrm{unexpected})$ and task relevance $(\Delta_{\mathrm{decoding}}=\mathrm{relevant}-\mathrm{irrelevant})$ conditions. That is, each trace represents the average difference in accuracy within one manipulation (e.g., expectancy) while ignoring the other (e.g., task relevance). ***d***, Decoding accuracy for the target location of expected targets, when tested on a decoder trained on unexpected targets, along the task-relevant plane. Horizontal bars in ***d*** indicate cluster-corrected periods when decoding accuracy differs significantly from chance (zero). Shaded areas in ***a****–**d*** indicate ±1 SEM; the bottom subpanel in ***c*** plots the corresponding Bayes factors (BF₁₀). We calculated the decoding accuracy difference $(\Delta_{\mathrm{decoding}}=\mathrm{expected}-\mathrm{unexpected})\;$ for each individual and correlated it with their behavioral precision difference $\Delta_{\mathrm{precision}}$ over time. Panel ***e*** schematically illustrates this analysis at a representative time point (224 ms; each blue dot, one participant). Panel ***f*** shows the resulting time-resolved correlations between neural and perceptual precision. The horizontal bar indicates cluster-corrected time windows where correlations were significant.

We applied inverted encoding modeling to reconstruct feature-specific neural representations of stimulus location for expected and unexpected trials to examine how expectancy and attention influence the neural fidelity of sensory events (Fig. 4*a*,*b*). We interpreted higher decoding accuracy as indicative of more precise neural representations, consistent with previous work decoding low-level stimulus features (Hu et al., 2025). We found a significant main effect of task relevance that extended from the beginning of the epoch (−0.2 s) to ∼0.13 s following stimulus onset, such that decoding accuracy was higher in the task-relevant condition (Fig. 4*c*). We also found a significant main effect of expectancy from ∼0.12–0.20 s and ∼0.27–0.33 s following stimulus onset, such that decoding accuracy was lower for expected targets. However, we found no significant interactions between task relevance and expectation.

The poststimulus expectation results (and the absence of an interaction with attention) mirror those found for recall precision, where expected stimuli were reproduced with poorer precision than unexpected stimuli. In contrast, the prestimulus effect of task relevance likely corresponds to the interaction between expectancy and task relevance observed for respond speed and accuracy; however, the rationale of this effect is less intuitive than that for expectancy. In the decoding analysis, trials were split by expectancy within each task relevancy condition. For both task-relevant and task-irrelevant conditions, expected and unexpected trial sets were matched in size via bootstrapping from the larger expected pool. Consider a brain signal that anticipates the target location, strongest when expectancy aligns with task demands (e.g., response priming). In task-relevant conditions, expected and unexpected trials are drawn exclusively from their respective task-relevant categories, so this signal correlates strongly (positively or negatively) with the true target location, supporting above-chance decoding in both cases. In task-irrelevant conditions, however, the expected and unexpected trial pools each contain a mixture of task-relevant expected and unexpected trials, diluting the correlation between the anticipatory signal and actual target location and thus reducing decoding accuracy. If this putative neural signal explained the prestimulus effect of task relevance, we would expect to observe negative decoding accuracy during this period if we were to train the decoder on the unexpected trial data and then test it on the expected data, which is precisely what we found (Fig. 4*d*).

### Neurobehavioral mapping

So far, we have found that expected events were both represented (neural) and replicated (perceptual) with reduced precision, independently of task demands. We further examined whether individual differences in the fidelity of expected and unexpected stimuli predicted recall precision (Fig. 4*e*,*f*) by leveraging independent estimates of neural and perceptual fidelity. For each participant, we computed the difference in decoding accuracy between expected and unexpected stimuli across time $\left(\Delta_{\mathrm{decoding}}\right)$. We then correlated this temporal stimulus-decoding profile with participants' recall precision differences $\left(\Delta_{\mathrm{precision}}\right)$. These neural–behavioral correlations revealed two adjacent periods of positive relationship between 200 and 300 ms poststimulus onset (*ρ*_max_ = 0.53, *p* = 2.3 × 10^−4^), such that participants with less precise neural representations of task-relevant expected targets also localized these stimuli less precisely. In contrast, we did not find a significant relationship between neural and perceptual precision across the task irrelevant plane. This may be due to reduced signal-to-noise along the task irrelevant plane, as attention reduces trial-to-trial variability of neural signals (Arazi et al., 2019). Indeed, the effect of expectancy on decoding accuracy was clearer along the task relevant plane. While expected targets were decoded significantly less accurately than unexpected targets across both task-relevant and task-irrelevant planes, only those across the relevant plane survived cluster correction.

## Discussion

For decades, researchers have sought to understand how the brain manages to process the vast, continuous stream of sensory inputs with such adaptive efficiency, despite its computational demands (Barlow, 2012). A prominent theoretical framework, predictive processing, attributes this efficiency to the brain's capacity to actively generate internal models that exploit statistical regularities developed across phylogenetic, ontogenetic, and proximate timescales (Rao and Ballard, 1999; Friston, 2005). The metabolic advantage of this predictive mechanism is often empirically observed as attenuated overall neural responses required for processing expected events (Summerfield et al., 2008; Kok et al., 2012a; Tran et al., 2021). One of the key questions, however, is whether this minimized energy cost arises from prioritizing sensory processing of expected (statistically frequent, and thus reliable), or unexpected (statistically infrequent, and therefore informative) events, as each could yield complementary, context-sensitive neurometabolic benefits (Yon et al., 2018; Press et al., 2020).

Extending recent evidence separately from neuroimaging (Tang et al., 2018; Richter et al., 2022) and behavioral (Lee and Rideaux, 2025; Rideaux et al., 2025b) works, here we found that unexpected events are prioritized over expected events in both neural and perceptual fidelity. Critically, we further revealed that this perceptual precision closely tracked neural representational fidelity within individuals, establishing a neurobehavioral link of how expectation shapes sensory fidelity. Comparison of responses to expected (high probability) and unexpected (low probability) events to those with no expectation (equiprobable) revealed that expected events are encoded with reduced fidelity, while unexpected and equiprobable events were treated equivalently. These findings are consistent with a dampening account of predictive processing; that is, the reduced neural responses associated with expected stimuli (expectation suppression) reflects suppression of neurons preferentially tuned to expected features. Unexpected events, where sensory inputs diverge from prediction, offer the greatest information gain for refining internal models of sensory inputs. Strategically allocating neurometabolic resources to these events therefore constitutes an adaptive redistribution of the brain's limited metabolic capacity—one that sustains more accurate future engagements with the environment (Ali et al., 2022). This selective prioritization of informative events resonates with the broader principle of efficient coding (Barlow, 2012), in which the brain economizes energy not by suppressing activity uniformly, but by comparatively directing it toward signals that yield the greatest potential for model updating.

While sensory representations seemed to prioritize unexpected events, we also found that expected targets were instead responded to faster and more accurately under time pressure. Further, this facilitation was amplified under attentional load, indicating its sensitivity to immediate contextual demands. These findings, consistent with recent work investigating neural adaptiveness across multiple temporal contexts and hierarchical cortical scales (Munn et al., 2024; Lee and Rideaux, 2025), thus suggest that the brain likely achieves adaptive efficiency through two complementary processes: (1) early (prestimulus) processing that supports rapid motor responses to expected events and (2) later (stimulus) processing that conserves metabolic expenditure by dampening sensory responses to expected events.

The functional complementarity observed in the current study partially aligns with recent theoretical and empirical evidence of a temporal-transition mechanism in predictive processing, in which early sensory responses reflect sharpening of expected events while later responses reflect dampening (Press et al., 2020; McDermott et al., 2026). However, we found no evidence of early sharpening, or sensory preactivation (Myers et al., 2015; Kok et al., 2017), of expected events; rather, our results point to a prestimulus process that speeds up motor responses to expected events that is mediated by attention, i.e., motor priming (Wolpert and Flanagan, 2001).

Given the conflicting—and at times null—findings in the predictive processing literature (den Ouden et al., 2023; Hu et al., 2025), it is important to clarify how our results situate within, and distinctly contribute to, some of its other ongoing debates. The contentious evidence in previous works may also partly reflect variability in how expectancy has been conceptualized and consequently manipulated across studies. In some cases, paradigms have failed to adequately control for critical confounds, which can fundamentally influence whether, and how, expectation effects are empirically observed (den Ouden et al., 2023, 2025; Westerberg et al., 2025). Studies that rigorously control for low-level adaptation confounds often report null expectation effects, downplaying the assumption that higher-level predictive mechanisms universally drive neural efficiency and instead proposing simpler processes, such as stimulus-selective habituation, as more parsimonious explanations (Rideaux et al., 2023; Feuerriegel, 2024; Mohan and Rideaux, 2025). Our paradigm directly addresses this confound by manipulating higher-order statistical relationships between stimuli (repetition vs alternation across a cardinal axis), which minimizes low-level adaptation via avoiding repeated stimulation at identical spatial locations. Simultaneously, robust expectancy was established through rapid, high-density stimulus exposure, confirmed with involuntary physiological measures.

The current experiment established sensory expectations by exposing participants to global spatiotemporal regularities. While isolating the effect of expectation in response to these global regularities was our primary focus, the influence of local spatiotemporal proximity (or lack thereof) in our data may have implications for the interpretation of effects associated with serial presentation of stimuli, e.g., adaptation. In particular, using the same experimental paradigm, with equiprobable target locations, we previously showed that local spatiotemporal proximity (repetition vs alternation) influences behavioral response time, accuracy, and recall precision (Lee and Rideaux, 2025). Here we replicated these findings for response time and accuracy; however, we found that spatiotemporal proximity had no effect on recall precision. This indicates that the introduction of global spatiotemporal biases mitigates or cancels the influence of local spatiotemporal proximity on representational fidelity. These findings support the explanation that local (1-back) spatiotemporal proximity effects reflect an expectation of sensory stability (Lee and Rideaux, 2025), which can be overridden by more pervasive global patterns, rather than adaptation, which is separable from prediction (Tang et al., 2018).

While adaptation confounds can challenge the mechanistic contributions of expectation effects, another critical factor complicating their empirical interpretation is the role of selective attention (Feldman and Friston, 2010; Lee and Rideaux, 2025). Both attention and expectation are conceptualized as higher-level modulatory mechanisms that guide lower-level sensory processing toward adaptively relevant inputs (Smout et al., 2019). This conceptual overlap thus raises an important, yet debated, question about their functional relationship: whether attention gates the influence of expectation (Alink and Blank, 2021; Bouwkamp et al., 2025)—by determining whether expectation exerts influences on sensory processing—or simply amplifies their effects (Smout et al., 2019). Using an orthogonal manipulation of attention and expectancy, we found that participants responded faster and more accurately to statistically reliable, expected events regardless of task demands. This suggests that attention is not required for expectation to exert its influence on processing that supports rapid responses to statistically likely events. However, the facilitation of response speed and accuracy for expected events relative to unexpected events was further enhanced when attention was engaged, indicating that attention likely amplifies, rather than initiates, this adaptive benefit. Similarly, for adaptive processing favoring statistically informative, unexpected events, representational precision advantages persisted independent of attentional demands. However, while their neural correlates were more prominent when attention was engaged, the reduction in recall precision for expected events was unchanged by task demands. Given the robust neurobehavioral correspondence observed for targets along the task-relevant plane and prior evidence linking attention to neuromodulatory control of cortical gain (e.g., signal-to-noise modulation; Feldman and Friston, 2010; Briggs et al., 2013), the relatively reduced expectancy effects on neural fidelity in the task-irrelevant condition might be explained by a reduction in the effective signal-to-noise ratio when attention was disengaged—partially obscuring underlying expectancy effects that were nonetheless manifested behaviorally. Overall, our results support the view that selective attention amplifies response speed benefits of expectation, while the mechanism serving sensory encoding benefits operates independently of task demands. This may mean that the mechanism supporting rapid interaction with the environment, e.g., motor priming, has a limited capacity and relies on top-down signals to achieve effective allocation, while the process optimizing sensory encoding of information rich events, i.e., dampening, operates in a massively parallel manner.

A limitation of the current study was the lack of an equiprobable (neutral) condition. While here we leveraged previously published data to substitute for this condition (Lee and Rideaux, 2025), our statistical power to detect differences between conditions was no doubt reduced by the individual differences between participants in the two experiments. However, despite this additional variance, we observed a clear pattern of results for all behavioral metrics. Due to the absence of EEG recordings in the previous study, we were unable to perform the same comparisons between neural responses to equiprobable, improbable, and probable targets. Given the neurobehavioral correlations observed, and the overall alignment between neural and behavioral phenomena observed here, it seems most parsimonious to infer that the same directionality effects between the two, i.e., reduced neural fidelity for expected stimuli rather than increased neural fidelity for unexpected stimuli. However, future neuroimaging studies can validate this inference by testing all three conditions within the same experiment.

In conclusion, our findings, together with recent evidence across temporal contexts and hierarchical cortical scales (Press et al., 2020; Teufel and Fletcher, 2020), indicate that adaptive efficiency may arise from two complementary mechanisms that differentially exploit environmental regularities: (1) rapid motor responses to expected events supporting timely interaction with the environment and (2) encoding prioritization of unexpected events reducing metabolic expenditure and refining internal models to optimize future predictions of the environment. The former mechanism appears to be amplified, but not gated, by the context-mandated selective attention, whereas the latter seems to operate independently of task demands.

## References

[B1] Ali A, Ahmad N, de Groot, E, van Gerven MAJ, Kietzmann TC (2022) Predictive coding is a consequence of energy efficiency in recurrent neural networks. Patterns 3:100639. 10.1016/j.patter.2022.100639 36569556 PMC9768680

[B2] Alink A, Blank H (2021) Can expectation suppression be explained by reduced attention to predictable stimuli? Neuroimage 231:117824. 10.1016/j.neuroimage.2021.117824 33549756

[B3] Arazi A, Yeshurun Y, Dinstein I (2019) Neural variability is quenched by attention. J Neurosci 39:5975–5985. 10.1523/JNEUROSCI.0355-19.2019 31152124 PMC6650992

[B4] Auksztulewicz R, Friston K (2016) Repetition suppression and its contextual determinants in predictive coding. Cortex 80:125–140. 10.1016/j.cortex.2015.11.024 26861557 PMC5405056

[B5] Barlow HB (2012) Possible principles underlying the transformations of sensory messages. In: Sensory communication (Rosenblith WA, ed), pp 216–234. Cambridge: The MIT Press. 10.7551/mitpress/9780262518420.003.0013

[B6] Bays PM, Catalao RFG, Husain M (2009) The precision of visual working memory is set by allocation of a shared resource. J Vis 9:7. 10.1167/9.10.7 19810788 PMC3118422

[B7] Bouwkamp FG, van Haren JJG, de Lange FP, Spaak E (2025) Dynamic competition between selective attention and spatial prediction during visual search (p. 2025.07.10.664103). bioRxiv. 10.1101/2025.07.10.664103

[B8] Briggs F, Mangun GR, Usrey WM (2013) Attention enhances synaptic efficacy and the signal-to-noise ratio in neural circuits. Nature 499:476–480. 10.1038/nature12276 23803766 PMC3725204

[B9] Brouwer GJ, Heeger DJ (2009) Decoding and reconstructing color from responses in human visual cortex. J Neurosci 29:13992–14003. 10.1523/JNEUROSCI.3577-09.2009 19890009 PMC2799419

[B10] Brouwer GJ, Heeger DJ (2011) Cross-orientation suppression in human visual cortex. J Neurophysiol 106:2108–2119. 10.1152/jn.00540.2011 21775720 PMC3214101

[B11] Buhmann Z, Robinson AK, Mattingley JB, Rideaux R (2026) Inverted encoding of neural responses to audiovisual stimuli reveals super-additive multisensory enhancement. eLife 13:RP97230. 10.7554/eLife.97230 41728936 PMC12928699

[B12] Chaumon M, Bishop DVM, Busch NA (2015) A practical guide to the selection of independent components of the electroencephalogram for artifact correction. J Neurosci Methods 250:47–63. 10.1016/j.jneumeth.2015.02.025 25791012

[B13] Delorme A, Makeig S (2004) EEGLAB: an open source toolbox for analysis of single-trial EEG dynamics including independent component analysis. J Neurosci Methods 134:9–21. 10.1016/j.jneumeth.2003.10.009 15102499

[B14] den Ouden C, Zhou A, Mepani V, Kovács G, Vogels R, Feuerriegel D (2023) Stimulus expectations do not modulate visual event-related potentials in probabilistic cueing designs. Neuroimage 280:120347. 10.1016/j.neuroimage.2023.120347 37648120

[B15] den Ouden C, Kashyap M, Kikkawa M, Feuerriegel D (2025) Limited evidence for probabilistic cueing effects on grating-evoked event-related potentials and orientation decoding performance. Psychophysiology 62:e70076. 10.1111/psyp.70076 40391524 PMC12090177

[B16] Feldman H, Friston K (2010) Attention, uncertainty, and free-energy. Front Hum Neurosci 4:215. 10.3389/fnhum.2010.00215 21160551 PMC3001758

[B17] Feuerriegel D (2024) Adaptation in the visual system: networked fatigue or suppressed prediction error signalling? Cortex 177:302–320. 10.1016/j.cortex.2024.06.003 38905873

[B18] Friston K (2005) A theory of cortical responses. Philos Trans R Soc Lond B Biol Sci 360:815–836. 10.1098/rstb.2005.1622 15937014 PMC1569488

[B19] Groppe DM, Urbach TP, Kutas M (2011) Mass univariate analysis of event-related brain potentials/fields I: a critical tutorial review. Psychophysiology 48:1711–1725. 10.1111/j.1469-8986.2011.01273.x 21895683 PMC4060794

[B20] Harrison WJ, Bays PM, Rideaux R (2023) Neural tuning instantiates prior expectations in the human visual system. Nat Commun 14:5320. 10.1038/s41467-023-41027-w 37658039 PMC10474129

[B21] Hu Z, Tran DMD, Rideaux R (2025) Multimodal evidence challenges the effectiveness of probabilistic cueing for establishing sensory expectations. Imaging Neurosci 3:IMAG.a.152. 10.1162/IMAG.a.152 PMC12441825 40970211

[B22] Jiang J, Summerfield C, Egner T (2013) Attention sharpens the distinction between expected and unexpected percepts in the visual brain. J Neurosci 33:18438–18447. 10.1523/JNEUROSCI.3308-13.2013 24259568 PMC3834051

[B23] Kok P, Jehee JFM, de Lange FP (2012a) Less is more: expectation sharpens representations in the primary visual cortex. Neuron 75:265–270. 10.1016/j.neuron.2012.04.034 22841311

[B24] Kok P, Rahnev D, Jehee JFM, Lau HC, de Lange FP (2012b) Attention reverses the effect of prediction in silencing sensory signals. Cereb Cortex 22:2197–2206. 10.1093/cercor/bhr310 22047964

[B25] Kok P, Mostert P, de Lange FP (2017) Prior expectations induce prestimulus sensory templates. Proc Natl Acad Sci U S A 114:10473–10478. 10.1073/pnas.1705652114 28900010 PMC5625909

[B26] Lee K, Rideaux R (2025) The influence of temporal context on vision over multiple time scales. eLife 14:RP106614. 10.7554/eLife.106614 41032362 PMC12488186

[B27] Maris E, Oostenveld R (2007) Nonparametric statistical testing of EEG- and MEG-data. J Neurosci Methods 164:177–190. 10.1016/j.jneumeth.2007.03.024 17517438

[B28] McDermott HH, de Martino F, Schwiedrzik CM, Auksztulewicz R (2026) Dissociable dynamic effects of expectation during statistical learning. eLife 13:RP103689. 10.7554/eLife.103689 41773829 PMC12956278

[B29] Mohan V, Rideaux R (2025) Energy efficiency and sensitivity benefits in a motion processing adaptive recurrent neural network. Neural Netw 191:107834. 10.1016/j.neunet.2025.107834 40651252

[B30] Mostert P, Kok P, de Lange FP (2015) Dissociating sensory from decision processes in human perceptual decision making. Sci Rep 5:18253. 10.1038/srep18253 26666393 PMC4678878

[B31] Munn BR, Müller EJ, Favre-Bulle I, Scott E, Lizier JT, Breakspear M, Shine JM (2024) Multiscale organization of neuronal activity unifies scale-dependent theories of brain function. Cell 187:7303–7313. 10.1016/j.cell.2024.10.004 39481379

[B32] Myers NE, Rohenkohl G, Wyart V, Woolrich MW, Nobre AC, Stokes MG (2015) Testing sensory evidence against mnemonic templates. eLife 4:e09000. 10.7554/eLife.09000 26653854 PMC4755744

[B33] Oostenveld R, Praamstra P (2001) The five percent electrode system for high-resolution EEG and ERP measurements. Clin Neurophysiol 112:713–719. 10.1016/s1388-2457(00)00527-7 11275545

[B34] Pelli DG (1997) The VideoToolbox software for visual psychophysics: Transforming numbers into movies. 10.1163/156856897X00366

[B35] Press C, Kok P, Yon D (2020) The perceptual prediction paradox. Trends Cogn Sci (Regul Ed) 24:13–24. 10.1016/j.tics.2019.11.003 31787500

[B36] Preuschoff K, ‘t Hart BM, Einhauser W (2011) Pupil dilation signals surprise: evidence for noradrenaline’s role in decision making. Front Neurosci 5:115. 10.3389/fnins.2011.00115 21994487 PMC3183372

[B37] Rao RPN, Ballard DH (1999) Predictive coding in the visual cortex: a functional interpretation of some extra-classical receptive-field effects. Nat Neurosci 2:79–87. 10.1038/4580 10195184

[B38] Richter D, Heilbron M, de Lange FP (2022) Dampened sensory representations for expected input across the ventral visual stream. Oxford Open Neurosci 1:kvac013. 10.1093/oons/kvac013 PMC10939312 38596702

[B40] Rideaux R (2024) Task-related modulation of event-related potentials does not reflect changes to sensory representations. Imaging Neurosci 2:1–13. 10.1162/imag_a_00266 PMC12290829 40800348

[B39] Rideaux R, West RK, Rangelov D, Mattingley JB (2023) Distinct early and late neural mechanisms regulate feature-specific sensory adaptation in the human visual system. Proc Natl Acad Sci U S A 120:e2216192120. 10.1073/pnas.2216192120 36724257 PMC9963156

[B41] Rideaux R, Bays PM, Harrison WJ (2025a) Reply to: “Model mimicry limits conclusions about neural tuning and can mistakenly imply unlikely priors”. Nat Commun 16:6229. 10.1038/s41467-025-60860-9 40623984 PMC12234690

[B42] Rideaux R, Dang P, Jackel-David L, Buhmann Z, Rangelov D, Mattingley JB (2025b) Violated predictions enhance the representational fidelity of visual features in perception. J Vis 25:14. 10.1167/jov.25.4.14 PMC12060052 40277426

[B43] Smout CA, Tang MF, Garrido MI, Mattingley JB (2019) Attention promotes the neural encoding of prediction errors. PLoS Biol 17:e2006812. 10.1371/journal.pbio.2006812 30811381 PMC6411367

[B44] Summerfield C, Trittschuh EH, Monti JM, Mesulam MM, Egner T (2008) Neural repetition suppression reflects fulfilled perceptual expectations. Nat Neurosci 11:1004–1006. 10.1038/nn.2163 19160497 PMC2747248

[B45] Tang MF, Smout CA, Arabzadeh E, Mattingley JB (2018) Prediction error and repetition suppression have distinct effects on neural representations of visual information. eLife 7:e33123. 10.7554/eLife.33123 30547881 PMC6312401

[B46] Tang MF, Kheradpezhouh E, Lee CCY, Dickinson JE, Mattingley JB, Arabzadeh E (2023) Expectation violations enhance neuronal encoding of sensory information in mouse primary visual cortex. Nat Commun 14:1196. 10.1038/s41467-023-36608-8 36864037 PMC9981605

[B48] Teufel C, Fletcher PC (2020) Forms of prediction in the nervous system. Nat Rev Neurosci 21:231–242. 10.1038/s41583-020-0275-5 32221470

[B47] Teufel C, Dakin SC, Fletcher PC (2018) Prior object-knowledge sharpens properties of early visual feature-detectors. Sci Rep 8:10853. 10.1038/s41598-018-28845-5 30022033 PMC6051992

[B49] Tran DMD, McNair NA, Harris JA, Livesey EJ (2021) Expected TMS excites the motor system less effectively than unexpected stimulation. Neuroimage 226:117541. 10.1016/j.neuroimage.2020.117541 33186721

[B50] Westerberg JA, et al. (2025) Sensory responses of visual cortical neurons are not prediction errors (p. 2024.10.02.616378). bioRxiv. 10.1101/2024.10.02.616378

[B51] Wilcoxon F (1945) Individual comparisons by ranking methods. Biom Bull 1:80–83. 10.2307/3001968

[B52] Wolpert DM, Flanagan JR (2001) Motor prediction. Curr Biol 11:R729–R732. 10.1016/S0960-9822(01)00432-8 11566114

[B53] Yon D, Gilbert SJ, de Lange FP, Press C (2018) Action sharpens sensory representations of expected outcomes. Nat Commun 9:4288. 10.1038/s41467-018-06752-7 30327503 PMC6191413

